# Contextual Factors and Motor Skills in Indigenous Amazon Forest and Urban Indigenous Children

**DOI:** 10.3389/fpubh.2022.858394

**Published:** 2022-04-25

**Authors:** Marcelo Gonçalves Duarte, Nadia Cristina Valentini, Glauber Carvalho Nobre, Rodolfo Novellino Benda

**Affiliations:** ^1^Department of Physical Education, Universidade Federal de Mato Grosso do Sul, Corumbá, Brazil; ^2^School of Physical Education, Physiotherapy and Dance, Universidade Federal do Rio Grande do Sul, Porto Alegre, Brazil; ^3^Federal Institute of Education, Science and Technology of Ceará, Canindé, Brazil; ^4^School of Physical Education, Universidade Federal de Pelotas, Pelotas, Rio Grande do Sul, Brazil

**Keywords:** child development, motor skills, ethnical groups, children, indigenous peoples

## Abstract

This study investigated the contextual factors, motor performance, and body mass index across indigenous land children, indigenous urban children, and non-indigenous urban children. A number of 153 children, both sexes (71 girls, 46.4%), from 8 to 10 years were assessed. The Test of Motor Gross Development-3 was utilized. Indigenous land children showed higher motor performance ($\eta {{}^{2}}_{\rho }$ = 0.37 and $\eta {{}^{2}}_{\rho }$ = 0.19 locomotor and object control, respectively) than indigenous urban children (*p* < 0.03) and non-indigenous urban children (*p* < 0.01); Indigenous urban children showed higher motor performance than non-indigenous urban children (*p* < 0.01). Body mass index was similar across groups ($\eta {{}^{2}}_{\rho }$ = 0,02; *p* = 0.15). Motor performance of indigenous land children was explained by the contextual factors that lead to a more active lifestyle, unsupervised free time, and play outside. In urban areas, behavior was similar, and although indigenous urban children kept some play tradition, it was not strong enough to be a protective factor for the motor performance.

## Introduction

Several studies have reported lower motor performance (MP) and a high prevalence of overweight and obesity among children across several countries [(1–7). This scenario may be explained, in part, by the contextual factors (3, 8–12). The lack of opportunities and parental support for motor practices and the inadequate physical spaces inside and outside the home negatively affect children's behavior toward sports and exercise, bodyweight status, and MP (13–16). Other vital factors related to lower MP included higher time spent in sedentary tasks and the lower participation in moderate to vigorous physical activities and restrict opportunities the motor practices experienced in the community in which they live (12, 17–22). Although a substantial body of research focuses on the relationship of several factors and MP in children, contextual factors are still underinvestigated. Besides, most of the research has been conducted in Western, Educated, Industrialized, Rich, and Democratic (WEIRD) countries. Less is known about developing countries and even less about diversity groups, such as indigenous children living in those countries.

In Brazil, 12.2% of the national territory is indigenous land (i.e., lands that indigenous people have the original right and whose demarcation was regulated by the Brazilian Federal Constitution); its concentration is highest in the legal Amazonas (54%) (23). Migration from the indigenous land to nearby cities is a current factor, considering the Sateré-Mawé people; about 1,000 live outside the indigenous land in cities and rural areas near the Andirá-Marau indigenous land (24). The determinants of the migration are related to the increasing contacts with the non-indigenous population, growing access to information, searching for work and better education, and the relative facility to move to cities that are getting close to the indigenous lands. These factors had led to the changes in the traditional indigenous way of living, leading to indigenous communities' economic, social, and cultural transformations (24). Indigenous children, across 23 countries, had poor health outcomes and were disadvantaged regarding access to health services, the elevated prevalence of inadequate height-for-age, and anemia [(25–31)]. Less is known about other health parameters and behavioral factors. Up to date, we have not found studies that investigated how the contextual factors may affect the MP in indigenous children.

For example, in the Brazilian Amazonas indigenous land villages, indigenous children tend to spend most of their free time playing outdoors without supervision and enjoying greater mobility and autonomy to explore the village houses and the neighborhoods (32–34). Boys and girls also participated in several activities that imitated the adult world. Boys endeavor to hunt small animals with toy weapons on the outskirts of the village and expeditions in the woods to find fruits. Girls had the autonomy to get together to play with dolls, model clay, and paint wood and clay crafts (35). So, it is plausible to consider that these contextual factors promote or reinforce appropriate weight status and motor development by participating in daily activities, promoting a more active lifestyle, enabling higher levels of physical activity (PA), physical fitness, and motor coordination.

Besides, the evidence of the relationship between an active lifestyle and higher PA levels, physical fitness, and motor coordination is reported in children (22, 36–38); however, these findings are not examined among indigenous children living in villages in the indigenous land and urban areas. Many indigenous families have left the indigenous land and lived in urban areas, the so-called urban indigenous (24); how this new kind of life changes their lifestyle and may affect health and motor components is unknown. The migration of indigenous people to urbanized areas is increasing due to the cities' progressive proximity to the indigenous land, the lack of health support and schools in the village in the indigenous land, and conflict with the leaders or community members (24, 39). The migration of indigenous groups to the cities may change their behavior due to the contextual factors experienced in the urbanized environment, reflecting changes in their lifestyle (24). For example, the free time and use of spaces previously allowed to indigenous children living in the indigenous land may not be the same in the urbanized areas.

Children's MP still needs to be investigated in the light of contextual factors, within a geographic region, the diversity in motor experiences, and the importance placed on learning motor skills within different cultures (9, 19, 40–42). Therefore, this study investigates the differences in MP, body mass index (BMI), and contextual factors across three groups of children–indigenous children living in indigenous land in the forest, indigenous urban children living since infancy in the city, and non-indigenous urban children, and if BMI and contextual factors were related to MP for those children, as a secondary objective, sex differences were also examined.

## Methods

### Participants

The study included 153 children, 8 to 10 years old (M_age_ = 8.26, SD = 0.48 years), and their families (parents or guardians) living in two Amazonas cities (Barreirinha and Parintins), Brazil. The inclusion criteria included: (1) being 8-to-10-year old indigenous and non-indigenous children; (2) attend to public school at the urban area or village; (3) proof of being Sateré-Mawé by presenting the registry of indigenous birth and the self-declaration of ethnicity; (4) parental declaration that children never resided in the urban space–for the indigenous children living in the village, that children lived in the city of Parintins since birth or 2 years old–for urban indigenous children, of non-indigenous heritage for non-indigenous children. In this study, no child showed motor impairments or any health issue that might interfere with their ability to perform the motor tests. This information was obtained by parental declaration.

The study was composed of three distinct groups: 43 indigenous land children (20 girls and 23 boys)–children living in the of Andirá-Marau lands of the Sateré-Mawé ethnic group in the Ponta Alegre village (Barreirinha city–state of Amazonas). The second group was composed of 46 indigenous urban children (22 girls, 24 boys) from the Sateré-Mawé ethnic group, living in an urban area of Parintins. The third group was formed by 64 non-indigenous urban children (29 girls, 35 boys) living in Parintins. The number of indigenous children living in indigenous land encompassed the entire children's population in this age group in the Ponta Alegre village; children attended the village school. In addition, indigenous and non-indigenous children residing in the city of Parintins were enrolled in public schools. This research project was approved by the University Ethics Committee and the National Research Ethics Council (protocol number 62705916.1.0000.5149). The parents provided informed consent and children verbal assent.

### The Context

The study was conducted, enrolling the Sateré-Mawé people. The Sateré-Mawé people, a population approximately of 8,500, are the part of the Tapajós-Madeira culture, living in the indigenous land of the Andirá-Marau, a region in the mid-Amazon River, located in the Maués, Barreirinha, Parintins, Itaituba, and Aveiro counties, on Amazonas and Pará states border. The Andirá-Marau lands hold 91 villages, approximately 1,600 families, with 7,502 inhabitants, along the main rivers, Madeira and Tapajós, and several streams across this area (43). The Sateré-Mawé people speak Setere–a language that integrates the linguist Tupi-Guarani family and maintains their language despite the three centuries of exposure to the national society and Portuguese language (44). Our study focused on the three groups of children: the Sateré-Mawé indigenous children living in the forest and an urban area, and the non-indigenous children from the same region.

The Sateré-Mawé indigenous children lived in the Ponta Alegre village in the Amazon Forest. The access to the village is *via* the Andirá River. The village has around 800 people and the largest migration of residents, mainly to Parintins, Barreirinha, and Manaus (45, 46). The monthly per capita income is approximately $\frac{1}{4}$ of the minimum wage in Brazil (near 57 dollars). The village has 127 wooden homes, with no running water and no electricity. The village has a diesel generator to provide electricity; however, there is hardly any fuel. When fuel is available, usually provided by visitors, the power is turned on for around 2 h daily. The water consumed is taken from the river with buckets and kept in pots. The eating habits of the families included fruits and vegetables obtained from the village plantations and the forest. Fish and hunted wild animals are also part of the diet. Only one adult is responsible for hunting for the whole village, with one apprentice, but other adults cooperate with hunting according to the animal size if necessary.

The village has a school–conducted strictly by indigenous teachers, an indigenous health basic unit, and a soccer field, where adults play soccer in the evenings. The families' routine starts around 6'o clock in the morning. Fathers go farming or fishing. The mothers prepare their children for school–the school has only the morning shift–and deal with the housework (i.e., go to the river to wash clothes, clean the house, and prepare lunch). Children came back from school at noon, had lunch with the family, and then spent the rest of the day playing around the village. At nightfall, the children return home for dinner and bedtime. At the age of 5, the children go to the school in the village; Portuguese lessons were provided. All children are bilingual; they speak Sateré and Portuguese.

Our study also focused on the indigenous children born in the village who left the village, with their families, as toddlers (2 years old) and grew up in Parintins. Parintins has the second-highest density in the state of Amazonas; the city is also located in the lower Amazon region, with a population estimated at 111,575 inhabitants and a family's per capita income near 1/2 of Brazilian minimum wage (around 100 dollars) (47, 48). The city has a median human development index (HDI 0.658) (49). The indigenous population of the Sateré-Mawé ethnic group living in Parintins (AM), a total of 512 people, reside in 86 houses with a monthly per capita income of around $\frac{1}{4}$ of Brazilian minimum wage. There are no specific urban indigenous neighborhoods in the city of Parintins. However, close to the sanitary indigenous special district of Parintins, on the banks of the Amazon River, there lived approximately 20 families; the rest of the urban indigenous families lived in the city's neighborhoods, mainly in the poorest ones.

**Figure 1:**
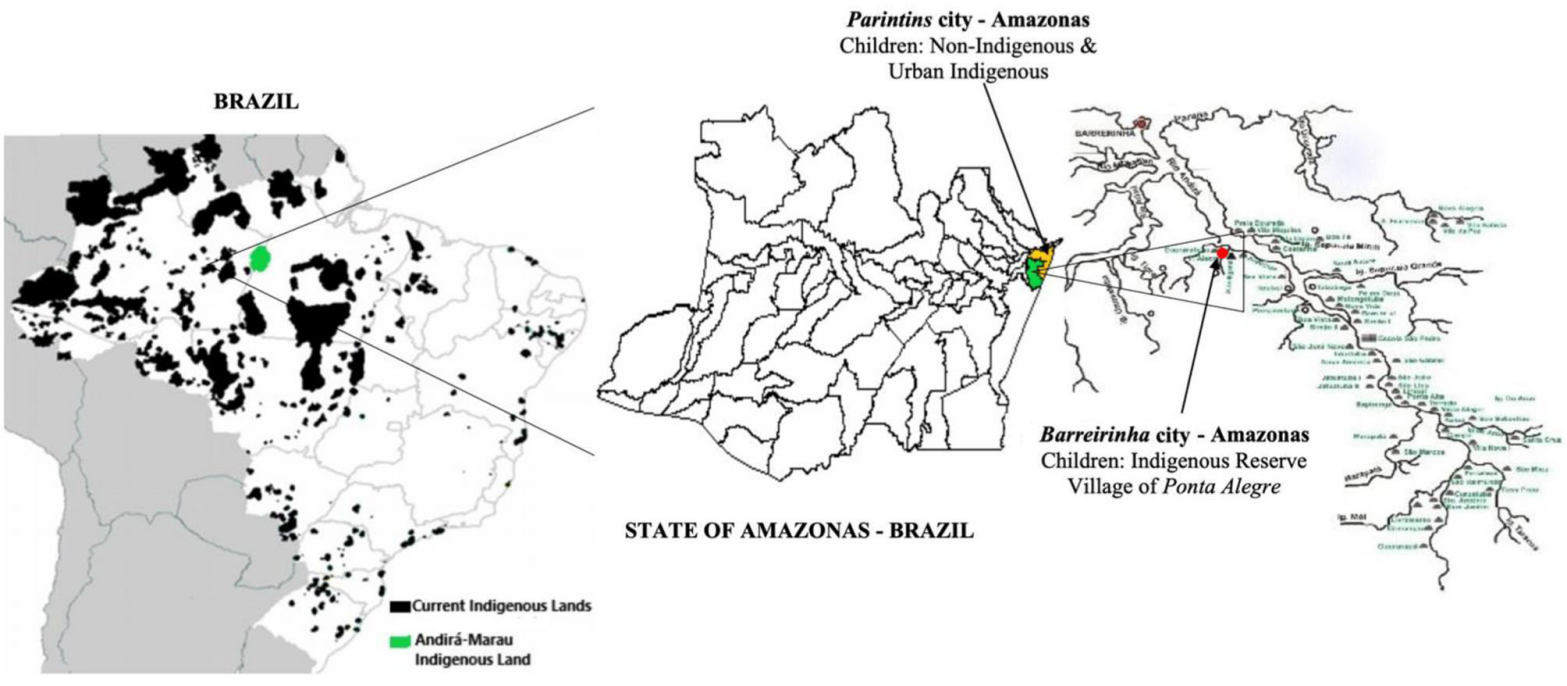
Brazilian map and the current Indigenous Lands in the Amazon Forest, Andirá-Marau Land, the geographic location of the Ponta Alegre village, and the city of Parintins–state of Amazonas.

Our study also focused on non-indigenous children. The non-indigenous children were born and raised in Parintins; they lived with their families in the poorest neighborhoods and attended the same public schools that the urban indigenous children attend. Figure 1 shows the Brazilian map with the current indigenous lands, the Andirá-Marau land, and the geographic location of the Ponta Alegre village and the city of Parintins.

### Measures and Assessment Procedures

#### Motor Performance

Motor Performance was measured with the Test of Gross Motor Development–Second Edition (TGMD-2) (50), validated in the Brazilian population (51). The TGMD-2 is a qualitative measure in which each skill is scored based on 3 to 5 criteria as present or absent; it has two subtests, locomotor skills (running, galloping, hopping, leap, horizontal jump, and sliding) and object control skills (striking a stationary ball, stationary dribbling, catching, kicking, overhand throwing, and underhand rolling). The test took ~20 min to administer for each child. Before each skill assessment, one demonstration was presented to the child, and then, the child performed one trial of practice and two actual trials. The assessments were conducted in an adequate and safe place with approximately 20 m in length and 9 m in width, following the test protocol (50). The assessments were conducted in an adequate and safe place with approximately 20 m in length and 9 m in width, following the test protocol (50). The assessments were video-recorded; two digital cameras were used (Sony Model DCR-SR 42, frequency of 60 hertz). One camera was positioned in front and another on the side to record the participants performing the locomotor and ball skills; the camera on the side was used to obtain additional information on ball skills. The videos were independently coded by three raters, experts in motor development and with 10 years of experience in motor assessment; high inter-rater reliability (intraclass correlation coefficient–ICC locomotor skills = 0.94, 95% CI: 0.91–0.96; ICC object control skills = 0.96, 95% CI: 0.93–0, 98) and intra-rater reliability for locomotor (ICC: Rater A = 0.81, 95% CI: 0.79–0.85; Rater B = 0.87, 95% CI: 0.84–0.91; Rater C = 0.89, 95% CI: 0.87–0.93) and object control skills (Rater A = 0.92, 95% CI: 0.87–0.94; Rater B = 0.93, 95% CI: 0.89–0.96; Rater C = 0.89, 95% CI: 0.87–0.93) were observed. In this study, we used the raw scores for locomotor and object control subtests (range from 0 to 48).

#### Body Mass Index

Body mass index was obtained by measuring the body mass (kg) and height (cm) following the World Health Organization guidelines (52). Body mass was determined on a Plenna® digital scale, scaled in kilograms, with an accuracy of 100 g. Height was obtained using an aluminum stadiometer (Balmak EST-222) with a 1 mm scale, ranging from 0 to 240 cm; BMI categorization was obtained according to the cutoffs established by the World Health Organization (52): severe thinness (< z-score −3); thinness (> z score −3 and < z score −2), eutrophic (> z score −2 and < z score +1), overweight (> z score +1 and < z score +2), obesity (> z score + 2 and < z score + 3), and severe obesity (> z score + 3).

#### Contextual Factors

A questionnaire was used to assess contextual factors; parents' responses were obtained from the interviews conducted by the principal researcher. Questions are related to the child's home environment, spaces, and free activities outside school, home, streets, and other places, such as the number of adults (2 or <2) and siblings at home (2, 3-to-4, <4); free time (>1, 1 to 2 h, <2 h); physical spaces to play (inside the home or out of home); sleep (> 9, 9 to 12, <12 h), television (no TV time, > 2 or <2 h), computer (no computer time, > 2 or <2 h) time; school commuting (non-motorized or motorized); playmates sex (same or opposite sex); and playmates age (younger, same age, older).

#### Procedures

The study was approved by the university and indigenous councils' ethical committee. To conduct the study in the indigenous land in the forest, the researchers contacted the Tuchaua (the Ponta Alegre village leader); the Tuchaua was responsible for authorizing the researcher's entry into the village and contacting the children's families. Then, a meeting was conducted with the families to inform the research goals, procedures, and assessment schedule. The schoolteacher, a native resident of Ponta Alegre village, supported the researchers during the meetings with the families and data collection, translating the information from Portuguese to Sateré language when necessary, facilitating the dyad with the native families. The researchers adjusted the study schedule to avoid the rain periods to collect the data in the Ponta Alegre village. They traveled by boat for 2 days and spent 5 days in the village.

We contacted the special indigenous sanitary district, a governmental healthcare agency for indigenous people, to mediate the contact with the urban indigenous residents in the city. For the non-indigenous children, the contact was initiated at the schools with the administrators. Information regarding the research goal, procedures, and schedules was provided for all families. Interviews and BMI were conducted at the residences, and the MP was conducted in the schools where children were enrolled. All parents signed the informed consent, and children verbally agreed to participate in the study.

#### Statistical Analysis

The measures of central tendency (mean: M), dispersion (standard deviation: SD), and confidence interval (95%CI) were used for MP (locomotor and object control) and BMI. Absolute frequencies and percentage were used for describing the children's weight status, and contextual factors. The models' assumptions–normal distribution (Shapiro–Wilk test values between 0.856 and 0.189), homogeneity of variances (Levene's test values between 4.232 and 1.223; *p*-values between 0.865 and 0.198), and the presence of significant outliers (visual inspection by boxplots, histograms, and scatterplots) were assessed. After checking the assumptions, the two-way ANOVA (group x sex) was used to compare locomotor and object control and BMI; Bonferroni's *post hoc* tests were used. The effect size was estimated using partial eta squared ($\eta {{}^{2}}_{\rho }$); values < 0.05 were adopted as a small effect, between 0.06 and 0.25 as a moderate effect, between 0.26 and 0.50 as high, and values > 0.50 as a very high effect (53, 54). Linear-by-linear association, with Bonferroni correction, were used to verify possible differences in the proportion of the categorical variables (weight status and contextual factors) among children's groups.

Univariate multiple linear regression analysis was used by the stepwise method, adopting that the last model was used to verify the associations between the BMI, contextual factors, and the MP (locomotor and object control). The models' assumptions, normal distribution, homogeneity, and independence of errors, were assessed. Normal distribution and homogeneity were assessed graphically, and the independence of errors was assessed using the Durbin–Watson test. The presence of outliers was assessed using the square distance of Mahalanobis (D^2^) and, when detected, was removed (54). Multicollinearity was assessed using the variance inflation factor (VIF) test; values greater than five were adopted as an indicator of the presence of multicollinearity (54). No indication of multicollinearity was observed. All analyses were conducted using the Statistical Package for Social Science (SPSS®), version 26. For all analyses, statistical significance was set at *p* < 0.05.

## Results

### Motor Performance, Body Mass Index, and Contextual Factor Comparisons Across Groups

**Figure 2:**
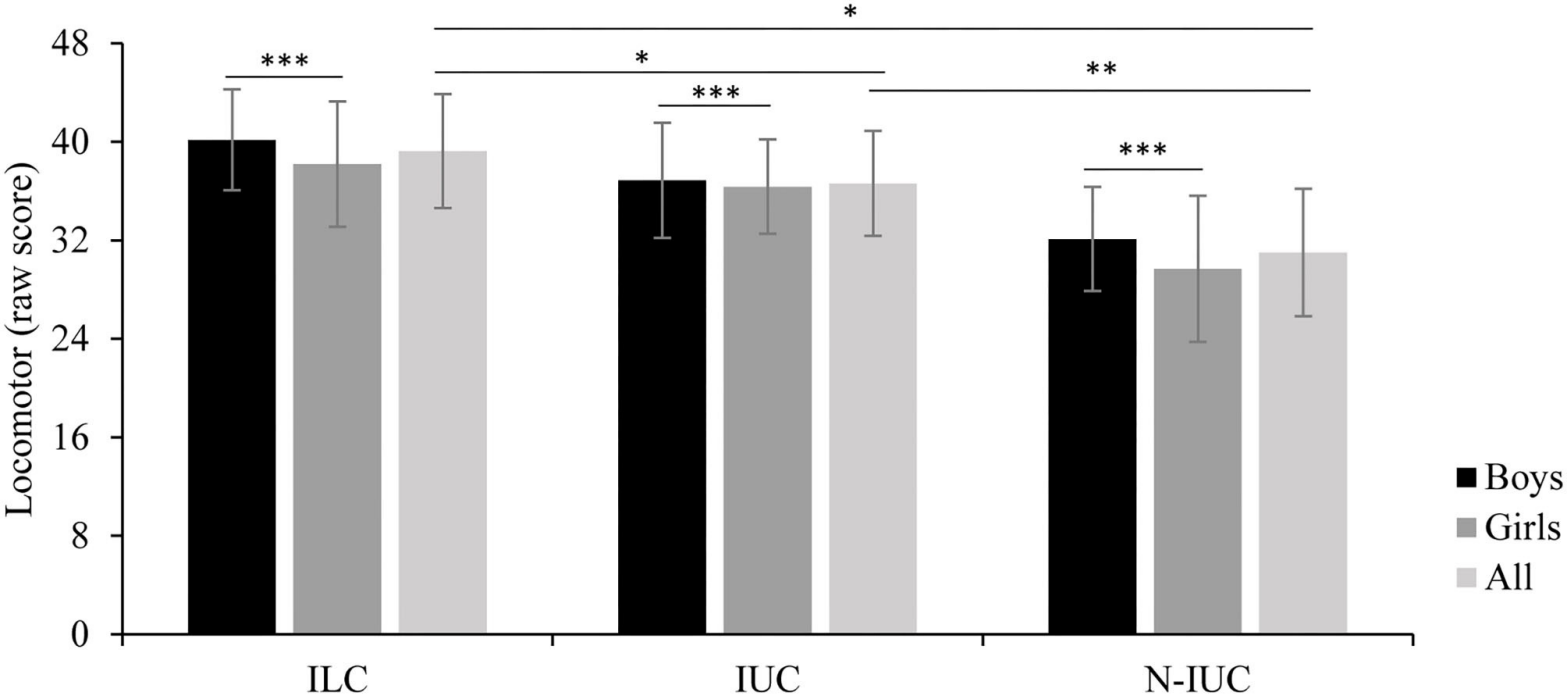
Locomotor scores by group; ILC, Indigenous Land Children; IUC, Indigenous Urban Children; N-IUC, non-indigenous urban children. Significant differences: *ILC > IUC (*p* < 0.001) and N-IUC (*p* < 0.001), **IUC > N-IUC (*p* < 0.015), ***All boys > All girls (*p* < 0.001).

**Figure 3:**
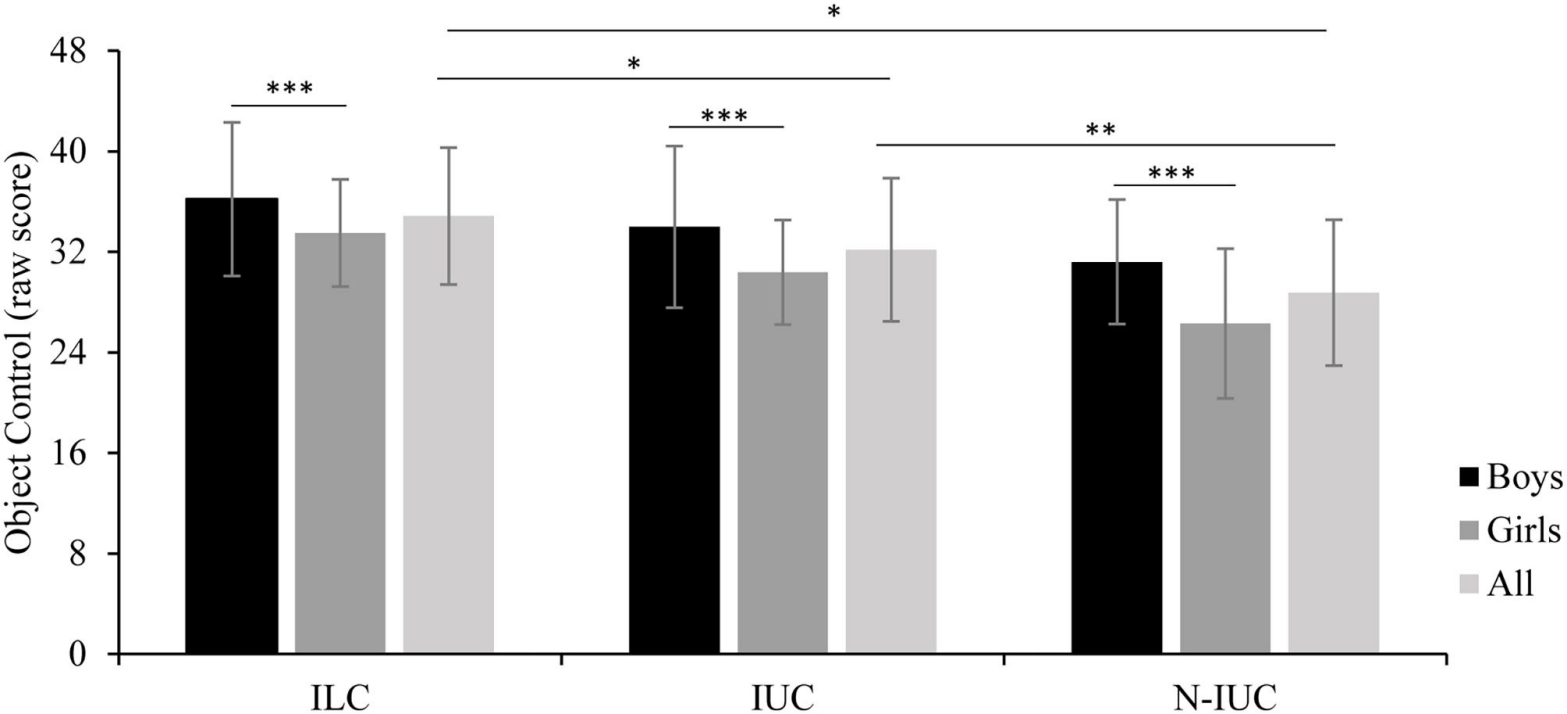
Object control scores by group; ILC, Indigenous Land Children; IUC, Indigenous Urban Children; N-IUC, non-indigenous urban children. Significant differences: *ILC > IUC (*p* = 0.019) and > N-IUC (*p* < 0.001), **IUC > N-IUC (*p* < 0.015), ***All boys > All girls (*p* < 0.001).

Figure 2 shows the locomotor scores and Figure 3 shows the object control scores by groups. The ANOVA showed a significant group effect for locomotor [*F*_(2,147)_ = 43.43, *p* < 0.001, $\eta {{}^{2}}_{\rho }$ = 0.37] and object control [*F*_(2,147)_ = 16.92, *p* < 0.001; $\eta {{}^{2}}_{\rho }$ = 0.19) skills. Indigenous land children showed higher motor scores than indigenous urban children (locomotor *p* = 0.034; object control *p* = 0.019) and non-indigenous urban children (locomotor *p* < 0.001; object control *p* < 0.001). Indigenous urban children showed higher scores than non-indigenous urban children (locomotor *p* < 0.001; object control *p* = 0.015). Significant sex effect was observed for locomotor [*F*_(1,147)_ = 4.44, *p* = 0.037; $\eta {{}^{2}}_{\rho }$ = 0.03] and object control [*F*_(1,147)_ = 18.87, *p* < 0.001; $\eta {{}^{2}}_{\rho }$ = 0.11] skills; boys demonstrated higher scores than girls.

Table 1 shows the BMI and contextual factor frequencies across the three groups. The ANOVA showed non-significant group [*F*_(2,147)_ = 1.88; *p* = 0.157; $\eta {{}^{2}}_{\rho }$ = 0,02] and sex [*F*_(1,147)_ = 0.33; *p* = 0.569; $\eta {{}^{2}}_{\rho }$ = 0.00] effects for BMI. The linear-by-linear association test indicated that BMI categorization did not significantly associate with groups (χ^2^ = 0.77, *p* = 0.380; Cramer's V = 0.18) and sex (χ^2^ = 0.04, *p* = 0.845; Cramer's V = 0.04).

**Table 1:** BMI and contextual factors of indigenous children living in the village, urban indigenous, and non-indigenous–N(%).

**Body mass index and** **contextual factors**			**Indigenous land children**			**Indigenous urban** **children**			**Non-indigenous urban children**		
			**Boys** **(***n*** = 23)**	**Girls** **(***n*** = 20)**	**All** **(***n*** = 43)**	**Boys** **(***n*** = 24)**	**Girls** **(***n*** = 22)**	**All** **(***n*** = 46)**	**Boys** **(***n*** = 35)**	**Girls** **(***n*** = 29)**	**All** **(***n*** = 64)**
BMI categorization		Severe thinness	3 (13)	0 (0)	3 (7)	2 (8.3)	2 (9.1)	4 (8.7)	1 (2.9)	5 (17.2)	6 (9.4)
		Thinness	3 (13)	1 (5)	4 (9.3)	3 (12.5)	3 (13.6)	6 (13)	1 (2.9)	4 (13.8)	4 (6.3)
		Eutrophic	16 (69.6)	19 (95)	35 (81.4)	14 (58.3)	10 (45.5)	24 (52.2)	33 (94.3)	15 (51.7)	47 (73.4)
		Overweight	1 (4.3)	0 (0)	1 (2.3)	2 (8.3)	2 (9.1)	4 (8.7)	0 (0)	5 (17.2)	5 (7.8)
		Obese	0 (0)	0 (0)	0 (0)	3 (12.5)	5 (22.7)	8 (17.4)	0 (0)	0 (0)	2 (3.1)
Contextual Factors	Adults at home*	2 adults	11 (47.8)	5 (25)	16 (39.5)	11 (45.8)	6 (27.3)	17 (37)	4 (11.4)	7 (24.1)	11 (17.2)
		> 2 adults	12 (52.2)	15 (75)	27 (60.5)	13 (54.2)	16 (72.7)	29 (63)	31 (88.6)	22 (75.9)	53 (82.8)^a^
	Siblings at home	2 siblings	3 (13)	–	3 (7)	4 (16.7)	2 (9.1)	6 (13)	6 (14.3)	7 (24.1)	12 (18.8)
		3 to 4 siblings	6 (26.1)	6 (30)	12 (27.9)	13 (54.2)	9 (40.9)	22 (47.8)	18 (51.4)	11 (37.9)	29 (45.3)
		> 4 siblings	14 (60.9)	14 (70)	28 (65.1)	7 (29.2)	11 (50)	18 (39.1)	12 (34.3)	11 (37.9)	23 (35.9)
	Unsupervised Free time*	<2 h	8 (34.7)	5 (25)	13 (30.3)	12 (50)	10 (45.5)	22 (47.8)	20 (57.1)	26 (89.7)	47 (71.9)^a^
		> 2 h	15 (65.3)	15 (75)	30 (69.8)	12 (50)	12 (54.5)	24 (52.2)	15 (42.9)	3 (10.3)	23 (28.1)
	Place to play*	Inside home	3 (13)	3 (15)	6 (14)	5 (20.8)	5 (22.7)	10 (21.7)	24 (68.6)	21 (72.4)	45 (70.3)^a^
		Out of home	20 (87)	17 (85)	37 (86)	19 (79.2)	17 (77.3)	36 (78.3)	11 (31.4)	8 (27.6)	19 (29.4)
	Sleeping time	<9 h	2 (8.7)	12 (60)	14 (32.6)	6 (25)	8 (36.4)	14 (30.4)	15 (42.9)	7 (24.1)	22 (34.4)
		9 to 12 h	10 (43.5)	7 (35)	17 (39.5)	16 (66.7)	7 (31.8)	23 (50)	15 (42.9)	15 (53.1)	30 (46.9)
		> 12 h	11 (47.8)	1 (5)	12 (27.9)	2 (8.3)	7 (31.8)	9 (19.6)	5 (14.3)	7 (24.1)	12 (18.8)
	Television time**	No time	23 (100)	20 (100)	43 (100)	5 (20.8)	0 (0)	5 (10.9)	0 (0)	0 (0)	0 (0)
		<2 h	0 (0)	0 (0)	0 (0)	11 (45.8)	2 (9.1)	13 (28.3)	9 (25.7)	6 (20.7)	15 (23.4)
		> 2 h	0 (0)	0 (0)	0 (0)	8 (33.3)	20 (90.9)	28 (60.9)	26 (74.3)	23 (79.3)	49 (76.6)^a^
	Computer time**	No time	23 (100)	20 (100)	43 (100)	10 (41.7)	8 (36.4)	18 (39.1)	6 (17.1)	4 (13.8)	10 (15.6)
		<2 h	0 (0)	0 (0)	0 (0)	11 (45.8)	13 (59.1)	24 (52.2)	13 (37.1)	5 (17.2)	18 (28.1)
		> 2 h	0 (0)	0 (0)	0 (0)	3 (12.5)	1 (4.5)	4 (8.7)	16 (45.7)	20 (69)	36 (56.3)^a^
	School commute	Non-motorized	23 (100)	20 (100)	43 (100)	6 (25)	9 (40.9)	15 (32.6)	13 (37.1)	4 (13.8)	17 (26.6)
		Motorized	0 (0)	0 (0)	0 (0)	18 (75)	13 (59.1)	31 (67.4)	22 (62.9)	25 (86.2)	47 (73.4)
	Playmates Sex	Same sex	20 (87)	18 (90)	38 (88.4)	21 (87.5)	20 (90.9)	41 (89.1)	26 (74.3)	20 (69)	46 (71.9)
		Opposite sex	3 (13)	2 (10)	5 (11.6)	3 (12.5)	2 (9.1)	5 (10.9)	9 (25.7)	9 (31)	18 (28.1)
	Playmates Age	Younger	9 (39.1)	1 (5)	10 (23.3)	1 (4.2)	3 (13.6)	4 (8.7)	12 (34.3)	10 (34.5)	22 (34.4)
		Same age	14 (60.9)	18 (90)	32 (74.4)	19 (79.2)	17 (77.3)	36 (78.3)	18 (51.4)	19 (65.5)	37 (57.8)
		Older	0 (0)	1 (5)	1 (2.3)	4 (16.7)	2 (9.1)	6 (13)	5 (14.3)	0 (0)	5 (7.8)

* *Significant differences across the three groups*;

** *Significant differences between the two groups: indigenous urban children and non-indigenous urban children–no variation was observed for indigenous land children, and therefore, they were excluded from the analysis; ^a^Significant higher proportion than the other groups*.

Regarding the contextual factors, the linear-by-linear test showed a significantly higher proportion of the non-indigenous urban children living with more than two adults at home [χ^2^ (1) = 5.66, *p* = 0.017, Cramer's V = 0.23], mainly the grandparents and uncles and aunts. In contrast, indigenous land children and indigenous urban children lived with at the maximum of two adults at home–their parents.

The analysis also showed a significantly higher proportion of the non-indigenous urban children that had <2 h of free time [χ^2^ (1) = 7.23, *p* = 0.007, Cramer's V = 0.36] and that the most prevalent space of playing was the inside home [χ^2^ (1) = 29.74, *p* < 0.001, Cramer's V = 0.53] compared to the indigenous children from the forest and the urban area. The activities in the free time were unsupervised for the three groups and involved similar games for the urban area. Indigenous urban children played kite, soccer, and run-and-catch games, and non-indigenous urban children played kite, soccer, and dodgeball, whereas indigenous land children usually played run-and-catch, balance games on lines, and games in the river. Although there was a soccer field in the Ponta Alegre village, the indigenous land children played very little soccer; the field and the ball were reserved for adult men. The inside home activities were similar across the three groups, the girls helped the mother with the home chores and the care of siblings, and the boys helped the fathers chop wood and clean fish.

The analysis also showed a significantly higher proportion of the non-indigenous urban children, compared to the indigenous urban children, with screen time–watching television [χ^2^ (1) = 4,685, *p* = 0.030, Cramer's V = 0.27] and using the computer [χ^2^ (1) = 22.01, *p* < 0.001, Cramer's V = 0.49], more than 2 h daily each device; indigenous land children had no screen time.

### Relationship Between Body Mass Index, Contextual Factors, and Motor Performance

Table 2 shows the linear regression results for BMI and contextual factors significantly associated with locomotor and object control performance across groups. For indigenous land children, the regression showed that play freely for more than 2 h daily positively explained 40% of the variance in locomotor scores [*F*_(1,42)_ = 29.06, *p* < 0.001, $r_{a}^{2}$ = 0.40]; for object control scores, 28% of the variance was explained by play freely more than 2 h daily and by playing outside [*F*_(2,42)_ = 9.04, *p* < 0.001, $r_{a}^{2}$ = 0.28].

**Table 2:** Linear regression: factors associated with locomotor and object control performance by groups.

**Groups**	**Factors**	***B***	***SE***	**β**	***t***	***p***
**Indigenous land children**						
Locomotor	Unsupervised Free time > 2 h^a^	6.43	1.19	0.64	5.39	<0.001
Object control	Unsupervised Free time > 2 h^a^	6.72	1.62	0.57	4.14	<0.001
	Place to play-outside home^b^	4.84	2.15	0.31	2.25	0.030
**Indigenous urban children**						
Locomotor	Adults at home > 2 adults^a^	−3.96	1.14	−0.45	−3.48	0.001
	Sleeping time 9 to 12 h^b^	−2.30	1.10	−0.27	−2.09	0.042
Object control	Adults at home > 2 adults^a^	−4.96	1.15	−0.42	−3.30	0.002
	Television time > 2 h^c^	−2.52	1.06	−0.30	−2.38	0.022
**Non-indigenous urban children**						
Locomotor	Siblings-3 to 4^a^	−3.86	1.48	−0.37	−2.60	0.012
	Siblings > 4^a^	−6.78	1.61	−0.63	−4.20	<0.001
	Computer time > 2 h^b^	−1.58	0.78	−0.23	−2.01	0.048
Object control	Computer time > 2 h^a^	−3.04	0.93	−0.38	−3.25	0.002

*Indigenous Land Children: ^a^reference category: free time <1 h; ^b^reference category: play inside home; Indigenous Urban Children: ^a^reference category: 2 adults; ^b^reference category: sleeping time <9 h; ^c^reference category: no TV time; Non-Indigenous Urban Children: ^a^reference category: 2 siblings, ^b^reference category: no computer time*.

For the indigenous urban children, the regression analysis showed that living with more than two adults at home and sleeping between 9 to 12 h negatively explained 23% of the variance of the locomotor scores [*F*_(2,45)_ = 7.92, *p* < 0.001, $r_{a}^{2}$ = 0.23]. For indigenous urban children, living with more than two adults at home and spending more than 2 h watching television negatively explained 30% of the variance in object control scores [*F*_(2,45)_ = 10.67, *p* < 0.001, $r_{a}^{2}$ = 0.30].

For non-indigenous urban children, the results showed that living with three or more than four siblings and spending more than 2 h on the computer negatively explained 30% of the variance in locomotor scores [*F*_(3, 63)_ = 10.21, *p* < 0.001, $r_{a}^{2}$ = 0.30] and stay on the computer for more than 2 h negatively explained 13% of the variance in object control scores [*F*_(1,63)_ = 10.59, *p* = 0.002, $r_{a}^{2}$ = 0.13].

## Discussion

This cultural study investigates the differences in MP, BMI, and contextual factors across three groups of children–indigenous children living in the Amazon Forest in the indigenous land, indigenous urban children living since infancy in the city, and non-indigenous urban children, and if BMI and contextual factors were related to MP for those children, as a secondary objective, sex differences were also examined.

Our main findings were the higher MP for the indigenous children living in the forest and that contextual factors were associated with MP. The children living in the Amazon Forest in the Ponta Alegre village showed higher MP than urban indigenous and non-indigenous children. The higher MP in locomotor and object control showed by indigenous land children was related to their way of living–they had daily unsupervised free time (more than 2 h) and spent this time playing outside the home. They also had no screen time daily as they have no television or computer.

In this unsupervised free time outside the home, indigenous land children explore the environment, move freely around all the houses in the village, and use the central village courtyard for their games. Children play and live among trees and rivers, and nature was a part of their daily life. Playing games, walking in the forest, following parents around, imitating parents' behaviors, and playing and bathing in the river are the daily activities observed in indigenous children living in the forest village, similar as reported in the previous study (55). Besides, in many ethnic groups, indigenous children spend most of their free time in playing; as an example, like our findings, Parakanã indigenous children spent most of their free time in playing outside (39, 56). Active leisure lifestyle has been reported as a health-protective factor among urban children; here, we provided the evidence for indigenous children living in the forest (56, 57). Therefore, we added new evidence to the present knowledge showing that MP is related to cultural beliefs for the indigenous children living in the forest.

Another major finding of this study was related to urban indigenous children. Urban indigenous children, even with the migration to the urban environment during infancy, still play unsupervised for more than 2 h outside than the non-indigenous urban children and had higher MP scores than non-indigenous urban children. It is plausible to assume that the indigenous children playing' routine was preserved by the indigenous parents even when living in the urban environment; the previous studies with the Sateré-Mawé people support their resilience to acculturation in urban environments (24). However, indigenous children in the urban area watch television and use computers, although the frequency was not as high as for non-indigenous urban children.

Moreover, although they spend time in playing outside, it was not related to MP, contrary to the children in the forest. More than two adults living at home, sleeping for long hours (9 to 12 h), and spending more than 2 h watching television were negatively related to MP. The number of adults at home has been referred to as a factor that can negatively influence children's motor development (58). The studies have found that, in large families, the home environment suggests being less stimulating for children due to sharing parents' attention and the limited space inside the house to do more active tasks (59–61). Adopting inactive lifestyles with sedentary activities, such as watching television, were previously related to the deficits in MP in non-indigenous children since it takes children away from vigorous activities to the authors' knowledge, it is the first study that provided evidence for urban indigenous children (22, 36, 38).

Regarding non-indigenous urban children, these children had the higher frequencies of more than two adults living in their homes, more time in sedentary activities, television and computer, and less time for play, and they play primarily inside the homes. Play inside the home seems to be a cultural characteristic of non-indigenous children who play in parks or outside the home for a short time and spend time inside in more sedentary activities, such as watching television and computers, a fact in this study for non-indigenous urban children (56, 57). Besides, the number of siblings at home and computer time was negatively related to MP; both factors were also previously related to lower scores in MP, like our findings (22, 38, 59, 61). Large families with many siblings prevent the parents from giving full attention to child development, providing stimulating activities, and promoting environment exploration; besides, sharing toys and physical space to be active are related to lower MP, and our results confirm this trend. Besides, nowadays, children have more access and time using computers and, consequently, are less involved in active body play, and in our results, it was related to lower MP (22, 38, 61, 62). This study results give support to the relationship between less active behavior and low MP in children, especially those who live in urban contexts.

There was no previous study that assessed MP and correlated contextual factors in indigenous (living in the forest and urban areas) and non-indigenous children to the authors' knowledge. Nevertheless, the previous studies suggested that PA levels were higher among indigenous adolescents than non-indigenous (56, 57). For example, Malta et al. (57) revealed a 22.5% higher prevalence of PA among indigenous adolescents compared to non-indigenous. Behaviors and attitudes differ concerning the sociocultural environment; as observed in the present results, the way of living in the forest was the primary influence in the avoidance of sedentary behavior, the children had no screen at home and a physical environment full of opportunities to explore, and they did explore unsupervised. The number of adults and siblings did not influence their MP, possibly because they have more freedom to explore the spaces of the house since they stay for a short time inside their home; that is, the time spent inside the residence was mainly used to help with some chores and sleep. If the environment affords adequate opportunities for the children can actively engage in a diversity of motor activities, consequently they will improve their MP. Our results supported this contention for indigenous children living in the forest (19, 41, 63).

We also analyzed the sex effect in MP. Independent of the groups, boys showed better MP than girls in locomotor and object control skills. Similar results have been found in several studies for non-indigenous low-income children [(6, 19, 64, 65)]. Vigorous motor activities and incentives to practice sports are often more vital for boys (6). Therefore, boys are generally more involved with the games that require gross motor components in wide physical spaces (e.g., ball games). On the other hand, girls engage more in stationary play that requires fine motor skills and verbal communication, such as playing with dolls, and it was not different in this study; girls were more required to help the mothers inside the home, and distinctive gender roles were observed in the inside home activities (6, 59, 66).

Urban children, indigenous or not, share a common factor they live in more crowded houses and use more screens; these factors were harmful to their MP. It is even more complex for the indigenous urban children; this was a change in the previous way of living, and although they enjoyed some unsupervised time, this freedom was not used to be outside in contact with nature but spent in front of screens. The lack of physical spaces that allow for outdoor play prevents children from developing motor skills. The results emphasize the need to implement public policy to create physical spaces in urban areas to support children's mobility and outdoor play, exposing children to active contexts that positively influence choices for more active activities in urban areas.

Regarding BMI, most of the indigenous children living in the forest village, urban indigenous children, and non-indigenous children (81.4, 52.2, and 73.4%, respectively) were eutrophic. Although no difference was found for weight status across groups, many indigenous children (forest village: 16.3%; urban area: 21.7%) were severely thin or thin. Although notably higher, these results are comparable to the previous data from indigenous Sateré-Mawé children from Amazonas, which identified 3.63% with thinness or severe thinness (67). Unfortunately, poverty is the underlying condition for this factor; as reported in the present study, Sateré-Mawé people's monthly income is meager, placing them in Brazil's poverty line. At the other end of the curve, urban indigenous children showed a high percentage of overweight and obesity (26.1%). As the present study results, data from the food and nutrition surveillance system −22.1% of overweight and obese indigenous children and previous research regarding weight status of indigenous children – showed this trend in increasing obesity [(33, 68); Fávaro et al., 2019; Ferreira et al., 2021; (67, 69, 70)]. Changes in dietary practices, more frequent consumption of caloric, inadequate, industrialized foods, and the adoption of a more sedentary behavior resulting from the migration of these indigenous children to urban areas may explain this scenario (71, 72).

## Conclusion

Contextual factors experienced by indigenous children living in the forest village were more favorable for adopting an active lifestyle that positively impacted MP. Associated factors with the MP of children in this study were different, demonstrating the strength of contextual factors on motor development across groups. Indigenous children in the village do not have screen time (TV and computer); therefore, they spend more time enrolled in free activities in contact with nature, and they have more space to play than their urban peers. On the other hand, urban children (indigenous or non-indigenous) have less free time, smaller play areas, and more screen time. The unlimited physical space in the forest and being free to explore the environment autonomously is a characteristic of the indigenous culture and a protective factor for MP. These characteristics cannot be exported to other cultures; each investigated group had cultural particularities and values, and social expectations within each investigated context. However, our results support the notion that urban children need to have more free time outside and, if possible, more contact with nature to become more motor proficient. Providing urban children with accessible and challenging experiences to explore their motor repertoire may improve their MP. Regarding indigenous children living in the forest, although they lived with fewer resources in the village, they had what was necessary to develop motor skills–free time and physical space. This study advances by providing a further understanding of a population that has been less investigated and with very restricted access and for a better understanding of contextual factors that promote MP in different sociocultural environments. Following these children for a long time to investigate whether the cultural factors affecting MP would change as different opportunities would be experienced seems to be a future direction to pursue. Understanding the value of indigenous children and parents' place in motor activities was a limitation of this study and a recommendation for future research.

## Data Availability Statement

The raw data supporting the conclusions of this article will be made available by the authors, without undue reservation.

## Ethics Statement

The studies involving human participants were reviewed and approved by Universidade Federal de Minas Gerais. Written informed consent to participate in this study was provided by the participants' legal guardian/next of kin.

## Author Contributions

MD and RB participated in the design of the study. MD participated in the design of the study and contributed to data collection. MD, RB, GN, and NV contributed to data reduction/analysis and interpretation of results. All authors contributed to the manuscript writing and have read and approved the final version of the manuscript and agree with the order of presentation of the authors.

## Funding

This research was funded by CAPES (Coordenação de Aperfeiçoamento de Pessoal deNível Superior) and CNPq (Conselho Nacional de Desenvolvimento Científico e Tecnológico).

## Conflict of Interest

The authors declare that the research was conducted in the absence of any commercial or financial relationships that could be construed as a potential conflict of interest.

## Publisher's Note

All claims expressed in this article are solely those of the authors and do not necessarily represent those of their affiliated organizations, or those of the publisher, the editors and the reviewers. Any product that may be evaluated in this article, or claim that may be made by its manufacturer, is not guaranteed or endorsed by the publisher.
